# Progress in Fevers

**Published:** 1902-04-12

**Authors:** 


### Progress in Fevers.

**Small-pox.** — *Vaccination and Revaccination.*— Speaking on modern methods of vaccination, Monckton Copeman[1] reviews the history of vaccination from the publication of Jenner's pamphlet in 1798 to the last Vaccination Act of 1898. The "Conscience Clause" of the latter has, he thinks, justified its existence. Stimulated by the recollection of some fatal cases of erysipelas following vaccination which occurred during his boyhood, Dr. Copeman turned his attention to the substitution of animal lymph for that transferred from arm to arm, and as the outcome of his researches he found that if the pulp of the vaccine vesicles of the calf was triturated with a 50 per cent. solution of pure glycerine in distilled water, all extraneous organisms were inhibited and destroyed after the lapse of about four weeks. Sims Woodhead points out that it is the non-sporing organisms which are eliminated in this way, the spore-bearing organisms being almost entirely unaffected,[2] and suggests incidentally that this may give a clue to the nature of the vaccine organism; it may be too small to be seen by microscopes of present power. Copeman, however,[3] considers the spore-bearing organisms likely to be present are of no pathogenic importance. In the Government preparation of lymph according to Copeman's directions,[4] healthy calves from three to six months old are used. The posterior surface of the abdomen is shaved and carefully cleansed, and then lightly scarified with a sharp scalpel dipped in glycerinated calf lymph. The calf is so stabled as to prevent injury to the vaccinated surface, and after 120 hours the lines of vesicles are disinfected and carefully scraped, and the vaccine pulp received in sterilised weighed bottles. After trituration alone and with the glycerine solution, it is stored for four weeks. Each week agar plate cultures are made. When these show no growth the lymph is drawn into capillary tubes and tested on children at the Animal Vaccine Establishment, the efficiency being gauged from the result. It is then distributed through the National Vaccine Establishment to the public vaccinators. Previous to any trial on children, the calf, after the skin surface has been gently dressed, is weighed and slaughtered, and the lymph only used after careful post-mortem inspection has shown the calf to be absolutely healthy. In the instructions to public vaccinators special stress is laid on the sterilising of skin and scarifier, and the proper protection of the vaccinated surface. The production of four good vesicles not less than half an inch apart is required if possible. Experience shows that less inflammation results from more well-separated vesicles than from fewer which coalesce. The permanent scarring after modern vaccina[illegible]n may be very slight. Probably the huge dee[illegible] scars which used to result were frequently due to infection with some other organism than that of vaccinia. Chaumier, of Paris,[5] emphasises the necessity of using really active lymph for vaccinating the calf. T. D. Acland[6] urges that lymph can be efficiently standardised as to potency by inoculating rabbits instead of children. This method is successfully adopted in Lille. F. W. Andrews[7] reports the result of revaccination among certain of the nursing and ward staff of St. Bartholomew's. Of 61 persons between 20 and 30 years of age 50 showed "perfect vaccinia," and eight showed "fair vesicles." The inference seems to be that infant vaccination leaves a large number of persons in the third decade of life very imperfectly protected against small-pox, and consequently urgently requiring revaccination. The decreasing power of primary vaccination after 20 is also borne out by the preliminary publication of the statistics of small-pox by the Metropolitan Asylums Board[8] for 1901. The 1,017 cases show a mortality of 24·28 per cent., which is too high owing to the exclusion of convalescent cases not then discharged. The mortality among vaccinated cases is 1·87 under 20, and 17·52 over 20 years. The mortalities for all ages among the vaccinated, unvaccinated, and doubtful cases are respectively 14·21, 50·52 and 65·08. During 1884-1900, 2,198 persons were employed at the small-pox hospitals, and 17,900 cases of small-pox admitted. Of the employés only 17 took the disease, of whom four escaped revaccination and 13 were not revaccinated till after they had joined the ship. All the others were re-vaccinated. Twenty-one persons employed on disinfecting work, most of them by the borough councils, and none of whom had been vaccinated since infancy, were admitted with the disease last year, a state of affairs considered highly discreditable to the London sanitary authorities.[9] Wynter Blyth[10] points out how by revaccination a small-pox epidemic could be arrested in a week. J. MacCombie[11] lays stress on the quality as well as number of the marks. Regarding as the standard of efficient vaccination not less than $\frac{1}{3}$ sq. inch of well foveated surface, he gives the mortalities as 2·7 per cent. for the efficiently vaccinated, 8·7 per cent. for the inefficiently vaccinated, and 45-50 per cent. for the unvaccinated. He bears strong testimony to the value of revaccination, as shown in hospital officials. Nurses and other officials would be difficult to obtain without this protection for small-pox hospitals. W. A. Bond[12] also points out the overwhelming evidence in favour of revaccination as shown in this section of the community. Prof. Hacius, of Geneva,[13] claims to have produced typical vaccina in a child with lymph obtained by passing variolous pus through a succession of calves, though he acknowledges the experiment generally fails. Copeman[14] has succeeded by first passing the pus of variola through one or more monkeys and then through calves. He thinks it probable that the cow-pox of Jenner's time was derived from the mild small-pox produced by *inoculation*, then so prevalent.

1 Lancet, Dec. 21. 2 Ibid., Jan. 18, p. 160. 3 Brit. Med. Journ., Feb. 1, p. 269. 4 Lancet, Dec. 21. 5 Brit. Med. Journ., Jan. 4, p. 31. 6 Lancet, Jan. 18, p. 160. 7 Ibid., Jan. 11. 8 Ibid., Jan. 18. 9 Ibid., p. 173. 10 Ibid., Feb. 15, p. 470. 11 Ibid., Dec. 28, p. 1797. 12 Ibid. 13 Ibid., Jan. 18, p. 160. 14 Ibid., Dec. 26.

